# Age-associated differences in triceps surae muscle composition and strength – an MRI-based cross-sectional comparison of contractile, adipose and connective tissue

**DOI:** 10.1186/1471-2474-15-209

**Published:** 2014-06-17

**Authors:** Robert Csapo, Vadim Malis, Usha Sinha, Jiang Du, Shantanu Sinha

**Affiliations:** 1 Department of Radiology, University of California, San Diego, CA, USA; 2 Department of Physics, San Diego State University, San Diego, CA, USA

**Keywords:** Intrinsic strength, Muscle composition, UTE imaging, IDEAL, Aging

## Abstract

**Background:**

In human skeletal muscles, the aging process causes a decrease of contractile and a concomitant increase of intramuscular adipose (IMAT) and connective (IMCT) tissues. The accumulation of non-contractile tissues may contribute to the significant loss of intrinsic muscle strength typically observed at older age but their *in vivo* quantification is challenging. The purpose of this study was to establish MR imaging-based methods to quantify the relative amounts of IMCT, IMAT and contractile tissues in young and older human cohorts, and investigate their roles in determining age-associated changes in skeletal muscle strength.

**Methods:**

Five young (31.6 ± 7.0 yrs) and five older (83.4 ± 3.2 yrs) Japanese women were subject to a detailed MR imaging protocol, including Fast Gradient Echo, Quantitative Fat/Water (IDEAL) and Ultra-short Echo Time (UTE) sequences, to determine contractile muscle tissue and IMAT within the entire Triceps Surae complex, and IMCT within both heads of the Gastrocnemius muscle. Specific force was calculated as the ratio of isometric plantarflexor force and the physiological cross-sectional area of the Triceps Surae complex.

**Results:**

In the older cohort, total Triceps Surae volume was smaller by 17.5%, while the relative amounts of Triceps Surae IMAT and Gastrocnemius IMCT were larger by 55.1% and 48.9%, respectively. Differences of 38.6% and 42.1% in plantarflexor force and specific force were observed. After subtraction of IMAT and IMCT from total muscle volume, differences in intrinsic strength decreased to 29.6%.

**Conclusions:**

Our data establishes that aging causes significant changes in skeletal muscle composition, with marked increases in non-contractile tissues. Such quantification of the remodeling process is likely to be of functional and clinical importance in elucidating the causes of the disproportionate age-associated decrease of force compared to that of muscle volume.

## Background

The aging process is accompanied by a progressive loss of muscle mass and strength. These symptoms, commonly referred to as sarcopenia [1], have been recognized as serious medical conditions, negatively affecting physical function, mobility and vitality at older age [2,3]. Several groups have demonstrated that the age-associated declines in muscle strength significantly exceed those in muscle mass, which reflects a deterioration of muscle quality. For example, longitudinal data from the Health ABC study obtained in more than 1600 elderly people [4] suggest that, over the age of 70 years, thigh muscle cross-sectional area decreases at a rate of ~1% per year, while the concomitant losses of knee extensor muscle strength may be 2–5 times greater. For the plantarflexor muscles, cross-sectional comparisons between young (~25 yrs) and older (~75 yrs) cohorts similarly indicate a ~20% decrease of Triceps Surae muscle volume, accompanied by a ~35-40% loss of muscle strength [5,6]. These results clearly demonstrate that the quality of skeletal muscles, defined as the capacity to generate force relative to muscle size (and sometimes also referred to as ‘intrinsic strength’ [7]), progressively deteriorates during the aging process, while muscle atrophy *per se* is but one reason for the decline in functional performance. In the light of these important findings, it is imperative to elucidate the reasons for the deterioration of intrinsic strength and, thus, explain the mechanisms of *dynapenia*[8], the dramatic muscular weakness in older age.

In addition to the well described changes in the neural drive to muscles, fiber type composition, muscle architecture and single-fiber specific tension (for an extensive review of the causes of age-associated deterioration of muscle quality see [9]), aging may bring about alterations of skeletal muscle composition, resulting from increased infiltration of both intramuscular adipose (IMAT) and connective (IMCT) tissues. Inclusion of IMAT and IMCT into the muscle, also referred to as *myosteatosis*[10], may negatively affect a muscle’s capacity to generate force by (i) displacing contractile materials, (ii) altering the elastic properties of skeletal muscles and, consequentially, the dynamics of contraction [11], and (iii) compromising the efficient transmission of contractile forces [12]. However, reliable *in vivo* quantification of the total IMAT and IMCT content, an imperative for modeling the force generated in terms of the above factors, is complicated, since the DXA-techniques most commonly applied to determine body composition are insensitive to intramuscular tissue heterogeneity [13].

Several earlier studies have used either CT [14,15] or standard MR [16,17] imaging to assess IMAT content but the results reported in these studies were based on the segmentation of single cross-sectional images, most commonly obtained in the middle of the muscle belly. Thus, these estimations rely on the debatable assumption that IMAT is distributed homogeneously along the muscle’s length. MRI-based studies were further complicated by challenges in data acquisition, since the sequences used were either susceptible to bias resulting from magnetic field inhomogeneities (*T*_*1*_-weighted sequences) or suffered from long acquisition times and low signal-to-noise ratios (SNR) and required complex post-processing procedures (Three-Point Dixon technique) [18]. The more recently developed sequence, Iterative Decomposition (of water and fat) with Echo Asymmetry and Least squares estimation (IDEAL) [19,20] facilitates the absolute quantification of IMAT in a robust and SNR efficient manner, but this technique has yet to be fully expanded into clinical routine use.

Intramuscular connective or collagenous tissue, which comprises the extracellular matrix, is critical for the transmission of force and for the passive elastic response of skeletal muscle. Its quantification however is equally problematic with routine MRI. Because of their solid or semi-solid character, the protons within connective tissues are rigidly bound, with a concomitant very short spin-spin relaxation time (*T*_*2*_ of few μs) in comparison to fluidic protons in the rest of the anatomy with *T*_*2*_’s in the order of several ms [21]. Thus protons in the semi-solid collagenous tissues have very little signal strength in routine MRI and appear as signal void. Only with very sophisticated and high-end hardware and data acquisition modifications of MRI can ultra-short echo time MR imaging be implemented which renders these connective tissues visible. Consequently, no imaging-based studies published to date have reported quantitative data of the IMCT network within skeletal muscles and its age-associated changes. Hence, reliable quantitation of the total amount of IMAT and IMCT and the age-associated differences in the relative quantities of these tissues is still outstanding.

The current study explores changes in muscle composition in cohorts of young (average age ~30 yrs) and older (>80 yrs) female participants. This is the first report of muscle composition in participants of this very older age, which necessitated tailoring acquisitions to accommodate the older subjects’ tolerance level. With this focus, the aims of the present study were to: (i) optimize a relatively rapid MRI protocol including water-saturated Fast Gradient Echo (FGRE) and Ultra-Short Echo Time (UTE) MR sequences and implement a Fuzzy C-Means algorithm for automated tissue segmentation, to objectively determine IMAT and IMCT content *in vivo*; (ii) apply these methods to the study of a cohort of young and older subjects to test the sensitivity of our approach in detecting age-associated differences in muscle composition; and, (iii) estimate the extent to which differences in the relative amount of IMAT and IMCT may affect a skeletal muscle’s intrinsic capacity to generate force.

## Methods

### Subjects

Five young (age: 31.6 ± 7.0 yrs, height: 155.7 ± 4.9 cm, mass: 48.4 ± 2.6 kg) and five older (age: 83.4 ± 3.2 yrs, height: 153.7 ± 1.5 cm, mass: 57.4 ± 4.9 kg) Japanese women were recruited via advertisements and from within retirement homes for Japanese people, respectively. All participants were free of internal or orthopedic disease. Written informed consent was obtained from all subjects. The study was approved by the Institutional Review Board of the University of California San Diego (071250) and conducted in agreement with the ethical principles for medical research outlined in the Declaration of Helsinki [22].

### MRI protocols

All MRI scans were performed on a 3 Tesla (T), GE scanner (General Electric Medical Systems, Milwaukee, WI, Ver. Signa HDxt). A custom-made (Millennial MRI Co., NY), 8-channel phased-array lower-leg coil system was used. Different combinations of the channels could be chosen to preferentially image a particular part of the lower leg with high SNR and excellent RF detection homogeneity over the typical 40 cm (maximum) field of view (FOV) used for the fat- and water-saturation sequences for morphology from the knee to the calcaneus. This large FOV avoided the extra time needed for repositioning of the coil that would be required if one used the smaller standard GE 8-Ch Knee coil. The signal homogeneity of the smaller ~20 cm FOV used for the UTE, DTI and IDEAL sequences was better than that of the standard GE knee coil.

**Figure 1:**
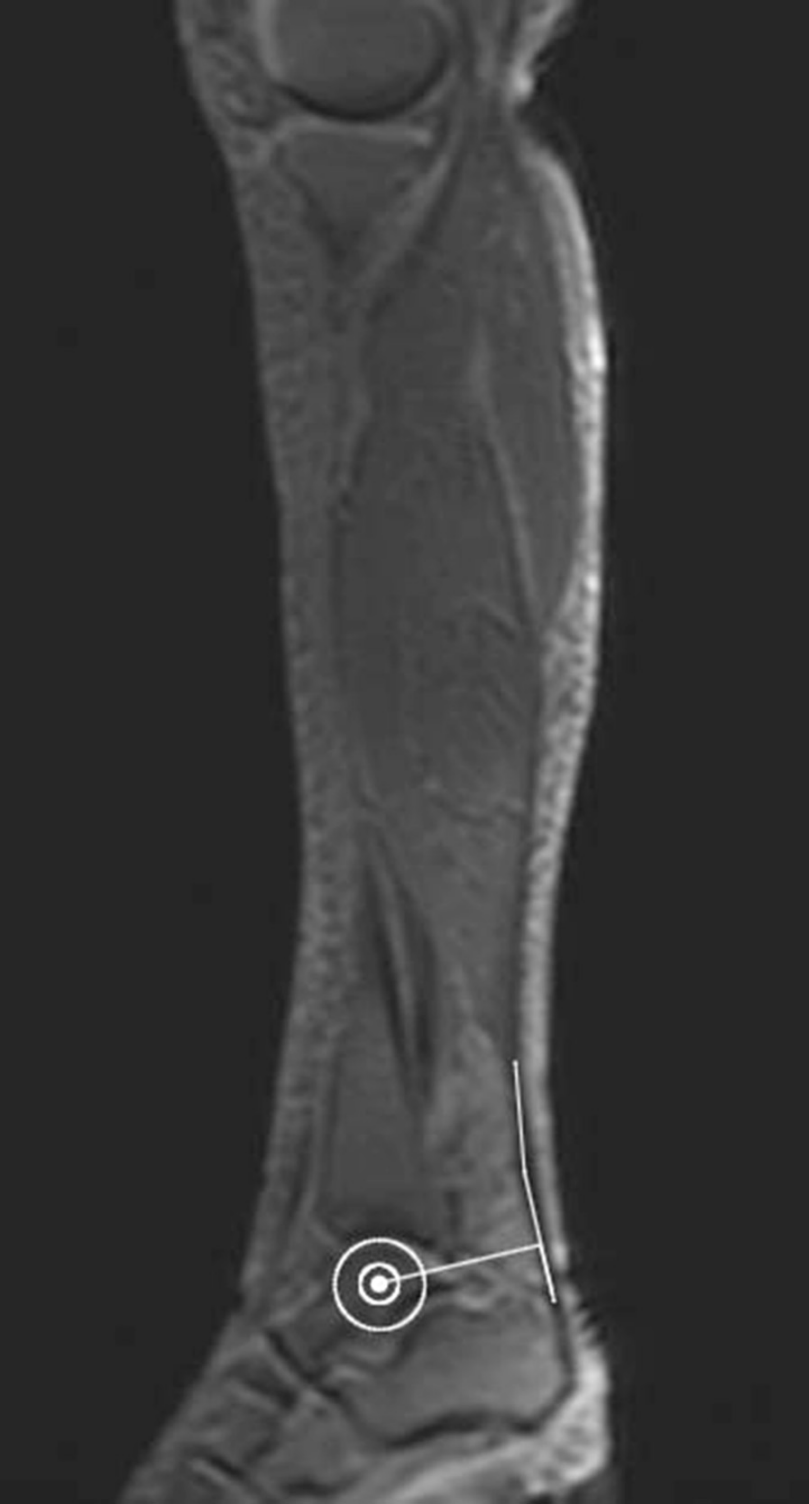
Sagittal-plane MR localizer used for measurements of Achilles tendon moment arm length.

(i) Following an initial 3-plane localizer, another Fast Gradient Recalled Echo (FGRE) was used to acquire several large FOV oblique sagittal images showing the anatomy from the origin of the Gastrocnemius Medialis muscle to the calcaneus. The (typically mid-sagittal) slice best showing the talus as well as the path of the Achilles tendon was used for measurement of the distances required to calculate the Achilles Tendon moment arm. For this purpose, a modified Reuleaux method [23] was used, assuming that the center of rotation of the ankle corresponds to the midpoint of a circle fitted around the talus (see Figure 1).

For the following sequences, smaller FOVs in the proximal part of the calf, covering either the Gastrocnemius muscles (UTE) or the entire Triceps Surae complex (other sequences) were used.

(ii) **Ultra-short echo time (UTE) Imaging:** Two sets of interleaved 2D UTE sequences were used with a short 40 to 80 μsec hard RF pulse for excitation followed by dual echo radial ramp sampling, FAST Gradient-echo acquisition. Long *T*_*2*_ signals were suppressed by echo subtraction. Sequence parameters included: FOV: 20 cm, TR = 200 ms, TE1 = 8 μs, TE2 = 2.6 ms, FA = 30°, BW = ± 62.5 kHz, NEX = 2, 256 × 256 half projections matrix, slice thickness of 5 mm with 5 mm gap, (with two sets of ~22 interleaving slices), scan time = 14 min for both scans.

(iii) **Morphologic Fat and Muscle (water) imaging:** Two sequences with the first for visualizing only muscles by suppressing fat (FatSat), and the second to visualize only fat, by suppressing water (WatSat), were acquired. These used FGRE, in-phase sequences with 2.1 TE, ~450 TR, 30° flip angle, 20 cm FOV, 5 cm thick with 0 mm spacing and typically 75 slices covering from the origin of Gastrocnemius muscle to the calcaneus, each sequence taking about 5 min.

(iv) **Quantitation Fat-Water (IDEAL) imaging:** In four subjects, including one participant from our younger cohort and three additional participants, a special sequence which yielded as the output separate fat and water images and enabled fat quantification, was made available. This sequence used the "iterative decomposition of water and fat with echo asymmetry and least-squares estimation" (IDEAL) method [20,24] combined with 3D gradient echo acquisition. This technique combines six asymmetrically acquired echoes with parallel imaging with an iterative least-squares decomposition algorithm to maximize noise performance. Unlike conventional fat-saturation methods, IDEAL is insensitive to magnetic field (B0 and B1) inhomogeneities and highly SNR-efficient [24,25]. The geometry parameters of the 3D gradient echo sequence matched those of the morphological imaging sequence; six TEs between 4.6 and 8.3 ms were used, the TEs used are specific to acquiring asymmetric echoes that are separated by 2π/3 with the middle echo at π/2+ π *k, k = 1,2; 5° FA; 3 echo train length; with 44 slices 5 mm thick in one 3D slab. The scan time for this one slab to cover the entire Gastrocnemius muscle was 5:20 minutes.

### MRI post-processing and image analyses

The outer contours of the Gastrocnemius Medialis (GM), Gastrocnemius Lateralis (GL) and Soleus (SOL) muscles were manually identified on a slice-by-slice basis using the fat-saturated FGRE series, since the muscles’ aponeuroses were most clearly visible on these images (Osirix Medical Imaging Software 5.6, Osirix, Atlanta, GA). Subsequently, the water-saturated FGRE images from the above protocol were cropped to remove subcutaneous fat, and 3D bias-field corrected by N4ITK Bias Correction [26] using publicly available image analysis software (3D Slicer 4.1, Harvard Medical School, Boston, MA). This step was performed to minimize intensity non-uniformities resulting from B1 (RF coil) inhomogeneity. The muscle contours were then superimposed on the pre-processed water-saturated FGRE images to create masked image stacks of GM, GL and SOL, respectively (MATLAB R2012b, Mathworks, Natick, MA). A publicly available Fuzzy C-means clustering algorithm (http://www.mathworks.com/matlabcentral/fileexchange/8351-fuzzy-c-means-thresholding[27]) was then applied to the masked images. 2D Fuzzy clustering was performed since this allowed the threshold to be set independently for each slice. A choice of three clusters yielded one cluster corresponding to muscle and fat each and an intermediate cluster with voxels including a combination of both tissues. The output of the Fuzzy clustering was threshold-segmented at an intensity value in the valley between the second and third cluster of intensity ranges. This empirical value of the threshold and other parameters of the algorithm (iterations: 100, level: 1∙10^-5^) were selected to yield IMAT values most closely matching the IDEAL fat fractions obtained for control purposes in four subjects, as described above. That is, the parameters of the Fuzzy clustering algorithm were tuned using the quantitative IDEAL images as the reference standard. It should be noted that the threshold was determined automatically for each image and provided consistent fat segmentation for all subjects, as evaluated by an expert.

The separation of ultra-short and long *T*_*2*_ tissues in the UTE images is challenging. One approach is to subtract the two imaging volumes acquired at the two different echo times: here, the first one at 8 μs and the second at ~3 ms. Since long *T*_*2*_ species will not decay significantly in this period, the subtracted image should ideally highlight only the short *T*_*2*_ tissue (i.e. IMCT). However, the segmentation of the subtracted UTE images proved difficult due to inadequate fat signal suppression and residual signal inhomogeneities. As an alternate strategy, *T*_2_^*^ maps were calculated to provide a robust means of non-contractile tissue segmentation: The time-dependent transverse magnetization (i.e. signal intensity) of each voxel is given by (1) *M*_*xy*_*(t) = M*_*0*_*e*^*iω0t*^*e(-t T*_2_^*-1^*)*, where *ω*_*0*_ is the Larmor frequency, *T*_2_^*^ is the effective tissue-specific transverse relaxation time and *t* is time. Substituting *t* with *TE*_*1*_ and *TE*_*2*_, equation (1) can be expressed as (2) *M*_*xy*_*(TE*_*1*_*) M*_*xy*_*(TE*_*2*_*)*^*-1*^*= e((TE*_*2*_*– TE*_*1*_*)T*_2_^*-1^*)*, which can be solved for *T*_2_^*^: (3) *T*_2_* = (*TE*_2_ - *TE*_1_) *ln*(*M*_*xy*_ (*TE*_1_) / *M*_*xy*_ (*TE*_2_))^- 1^. The *T*_2_^*^ of muscle/IMAT/IMCT tissue in the calculated maps was in the range 14-19/4-6/2-4 ms, respectively and provided an effective means of thresholding muscle from non-muscle voxels. A value of 8 ms was empirically determined to include all full and most partial volume non-muscle voxels. This threshold was applied across all subjects and provided consistent segmentation of non-muscle voxels, as evaluated by an expert. To evaluate the validity of the automated, threshold-based segmentation, the relative amounts of muscle and non-muscle voxels determined in one slice, coinciding with the greatest cross-sectional area of the Triceps Surae complex, were compared against those obtained through manual segmentation of the same image. The results of this comparison revealed very good agreement of the results obtained by automated and manual segmentation (r^2^ = 0.86, p < 0.001), with a mean difference of the content of non-contractile tissue of 4.3 ± 4.9% (larger ratios identified through automated segmentation). It must be noted that the *T*_2_^*^ of fat is low (in contrast to *T*_*2*_ of fat) due to the interaction between the different hydrogen nuclei within the fat molecule which results in a shortening of *T*_2_^*^. This resulted in a significant overlap of the *T*_2_^*^ histograms of IMAT and IMCT, complicating the direct separation of these two tissue types. Therefore, in a second step, the IMAT voxels identified from the water-saturated images were removed from the segmented *T*_2_^*^ maps (sum of non-muscle voxels) to obtain fat-free IMCT maps. The IMAT and IMCT maps clearly identified that fat infiltration tracks the connective tissue (spatial proximity). In order to quantitate this, the percentage of IMAT voxels with a connective tissue voxel in a 3 × 3 × 3 neighborhood was determined. The total volume of subcutaneous fat was also determined from the image stack.

### Measurements of muscle strength

To measure the strength of the plantarflexor muscles, the foot of the dominant leg, defined as the leg preferentially used to kick a ball, was strapped to a custom-made foot pedal device [28], with the knee fully extended and the ankle immobilized at 10° of plantarflexion (0° representing a right angle between the axis of the foot and the lower leg). The ball of the foot rested against a carbon-fiber plate to which an optical Fabry-Perot interferometer-based pressure transducer was glued. Pressure exerted on the carbon-fiber plate during contraction produced changes in the length of the optical cavity of the transducer which were detected and converted into voltages by a spectrometer (Luna Innovations Inc., Roanoke, VA), recorded using an indigenously-built LabVIEW module (NI LabVIEW 2011, National Instruments Corporation, Austin, TX, USA) and stored on a laptop computer. After five familiarization trials performed at submaximal intensity, all subjects executed three maximum voluntary contractions (MVC) over 5 s, interspersed by ~1 minute of passive recovery. The plantarflexion MVC strength was defined as the single highest point on the resulting voltage-time curve. The system was calibrated using disc weights to convert the recorded voltages into measures of torque (Nm). Subsequently, Achilles tendon forces were estimated from the equation *F* = *T MA*^- 1^, where *T* is the MVC plantarflexion torque and *MA* is the Achilles tendon moment arm length determined from sagittal-plane MR images [29]. To obtain a parameter of muscle quality, specific forces were calculated by dividing force by the total physiological cross-sectional area (PCSA) of the Triceps Surae complex. For this purpose, estimates of PCSA were obtained for each muscle from the equation *PCSA* = (*Vol cosθ*) *Lf*^- 1^, where *Vol* is muscle volume, *θ* the fascicle pennation angle, defined as the angle enclosed by the fascicle and the deep aponeurosis [30], and *Lf* fascicle length. Values of *θ* and *Lf* were determined in the distal and central muscle region by MR Diffusion Tensor Imaging-based fiber tracking, as performed in an independent study [31] on the same subjects. Subsequently, these muscle region-specific values were averaged for the calculation of PCSA. It should be noted that in this latter study, muscle architecture was only reported for the GM and GL muscles. To determine PCSA in the entire Triceps Surae complex, fiber tracks of the SOL were additionally obtained for the present study. The resulting values of *Lf* and *θ* were 5.4 ± 2.1 cm and 3.8 ± 1.0 cm as well as 24.0 ± 2.8° and 28.4 ± 7.6° in the young and senior cohort, respectively.

### Statistical analyses

The distributional normality of all data was tested by Shapiro-Wilk tests. Accordingly, independent-sample t-tests or Mann–Whitney U-tests were used to investigate between-group differences. Pearson’s correlation coefficients were calculated for correlational analyses and, for group comparisons, as estimates of effect size (t- or Z-transformation). The level of significance was set at α = 0.05 and data are reported as means ± SD. All statistical tests were carried out using SPSS Statistics 21.0 for Mac OS (SPSS Inc., Chicago, IL).

## Results

**Figure 2:**
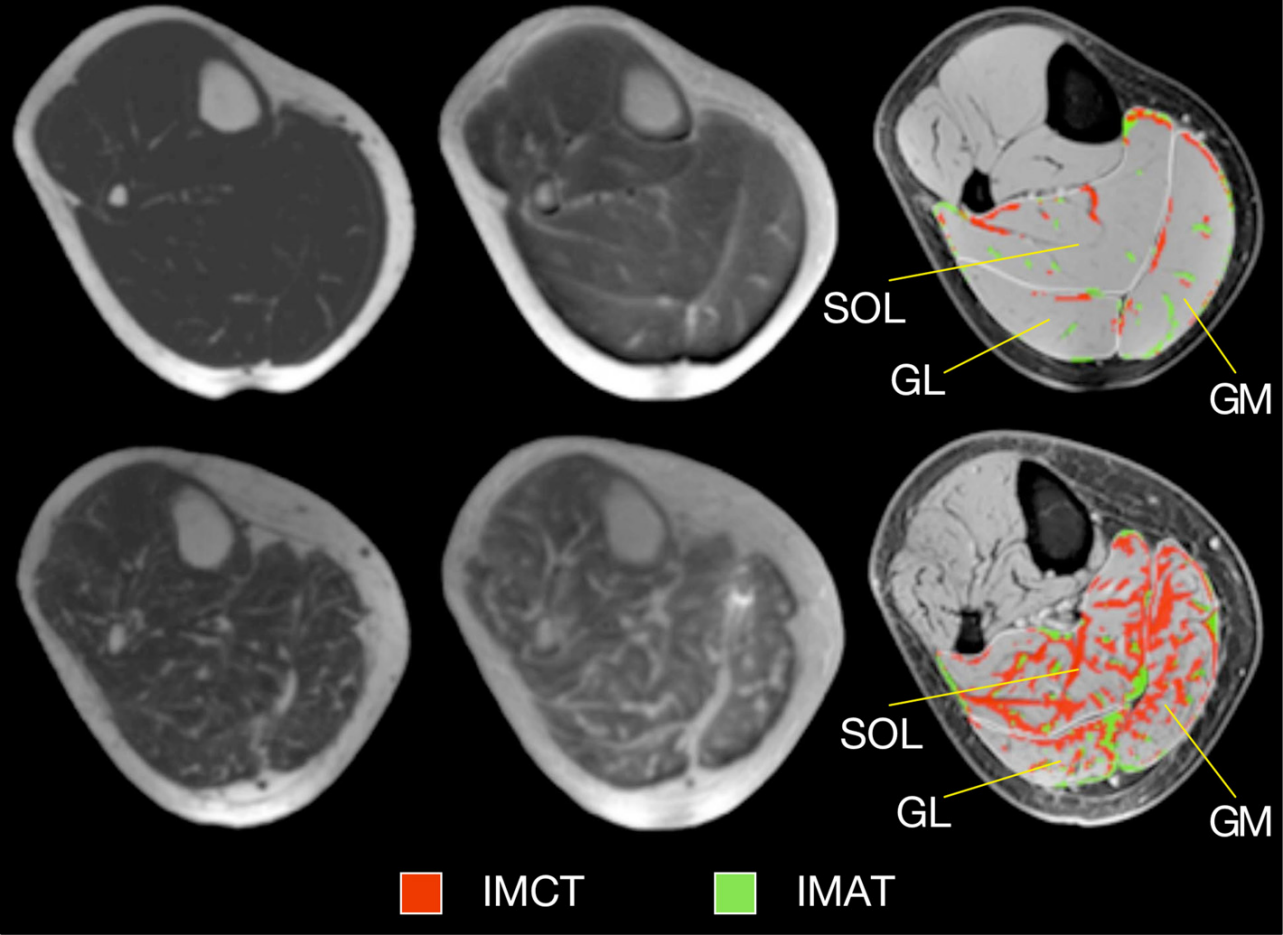
**Typical examples of MR images and resulting tissue segmentation in young and older women.** Left: Water saturated FGRE (showing IMAT), Middle: UTE (showing IMCT), Right: Standard morphological images with superimposed outer contours of muscles and the result of the automated tissue segmentation. Images in top and bottom row represent one young and old subject, respectively.

The results of the measurements of muscle size, force and specific force are summarized in Table 1. In the elderly cohort, Triceps Surae muscle volumes were smaller by 17.5%. Representative cross-sectional water-saturated FGRE (used for the segmentation of IMAT), UTE (for IMCT) and fat-saturated FGRE images with the superimposed results of the segmentation process (showing the amount and spatial distribution of IMAT and IMCT within the Triceps Surae complex) are displayed in Figure 2.

**Table 1:** Triceps Surae muscle volumes, force and specific force in young and older cohort

	**Young (n = 5)**	**Older (n = 5)**	***p *** **value**	**Effect size r**
GM Vol (cm^3^)	157.3 ± 32.2	121.2 ± 21.3	0.070	0.67
GL Vol (cm^3^)	74.6 ± 14.4	66.0 ± 9.1	0.293	0.37
SOL Vol (cm^3^)	307.8 ± 19.4	265.4 ± 47.2	0.119	0.55
TS Vol (cm^3^)	539.6 ± 63.9	452.6 ± 67.9	0.070	0.59
GM PCSA (cm^2)^	26.4 ± 5.0	31.9 ± 6.7	0.180	0.42
GL PCSA (cm^2^)	15.4 ± 2.2	17.0 ± 6.9	0.641	0.15
SOL PCSA (cm^2^)	57.4 ± 20.6	62.3 ± 17.7	0.702	0.13
TS PCSA (cm^2^)	106.0 ± 15.4	122.2 ± 32.1	0.451	0.31
Tendon Force (N)	1 426.3 ± 595.1	964.6 ± 319.3	0.165	0.48
Spec Force 1 (N/cm^2^)	14.74 ± 6.71	9.61 ± 5.35	0.218	0.39
Spec Force 2 (N/cm^2^)	17.88 ± 7.85	13.27 ± 7.56	0.372	0.29

GM: Gastrocnemius Medialis, GL: Gastrocnemius Lateralis, SOL: Soleus muscle, TS: Triceps Surae, PCSA: Physiological cross-sectional area. Spec Force 1: Specific Force as the fraction of force and PCSA as calculated from the total Triceps Surae volume. Spec Force 2: Specific Force as the fraction of force and PCSA as calculated from Triceps Surae volume after subtraction of intramuscular fat and connective tissues. Data are presented as means ± SD.

**Figure 3:**
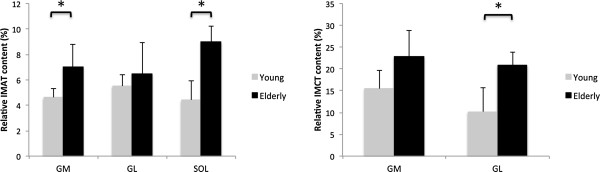
**Relative amount of Intramuscular adipose (left) and intramuscular connective tissue (right).** GM: Gastrocnemius Medialis, GL: Gastrocnemius Lateralis, SOL: Soleus muscle. Note: Significant between-group differences are highlighted with *.

In absolute terms, the IMAT contents measured in the young and older cohort were similar in the GM (young: 7.46 cm^3^ ± 2.49 cm^3^ vs. older: 8.74 cm^3^ ± 3.69 cm^3^, p = 0.538, r = 0.22) and GL (young: 4.06 cm^3^ ± 1.05 cm^3^ vs. older: 4.36 cm^3^ ± 2.07 cm^3^, p = 0.781, r = 0.10) but significantly larger by 57.7% in elderly subjects in the SOL muscle (young: 13.26 cm^3^ ± 3.98 cm^3^ vs. older: 24.01 cm^3^ ± 6.56 cm^3^, p = 0.14, r = 0.74). To validate our approach to determine IMAT contents, fat fractions were additionally determined by IDEAL measurements in four subjects. Correlational analyses demonstrated good agreement between results (r = 0.94), with the average difference (mean of GM, GL and SOL) of IMAT contents determined from water-saturated FGRE and IDEAL images being 1.38 cm^3^ ± 0.96 cm^3^. After normalization to muscle volumes, IMAT contents were found to differ significantly in the GM (p = 0.041, r = 0.76) and SOL (p = 0.001, r = 0.88) muscles (Figure 3, left chart) but not in the GM (p = 0.395, r = 0.30). In the entire Triceps Surae complex, IMAT contents tended to be larger in absolute terms (young: 24.78 ± 3.91 cm^3^ vs. older: 37.11 ± 11.59 cm^3^, p = 0.075, r = 0.71) and were significantly greater in the elderly cohort after normalization to muscle volumes (young: 4.6% ± 0.8% vs. older: 8.1% ± 1.4%, p = 0.001, r = 0.86). The neighborhood analysis for fat voxels revealed that, in young subjects, 62.3% ± 8.5% had a connective tissue voxel in their 3 × 3 × 3 neighborhood, while this value was significantly higher in the older cohort (86.3% ± 2.01%, p = 0.008, r = 0.83).

Due to time constraints in the data acquisition, IMCT contents could only be determined in the GM and GL muscles. Here, between-group comparisons revealed that IMCT contents were similar in the GM (young: 24.45 cm^3^ ± 8.21 cm^3^ vs. older: 28.32 cm^3^ ± 11.59 cm^3^, p = 0.560, r = 0.21) but significantly larger in the GL of elderly women (young: 7.72 cm^3^ ± 5.07 cm^3^ vs. older: 13.78 cm^3^ ± 2.91 cm^3^, p = 0.049, r = 0.63), representing a 56.4% difference in GL IMCT content. Reflecting these findings, normalization to muscle volumes revealed that the relative content of IMCT was significantly larger in the GL (p = 0.005, r = 0.80) with a further trend towards greater IMCT content in the elderly in the GM (p = 0.058, r = 0.62). The relative Gastrocnemius IMCT contents are depicted in Figure 3 (right chart). Jointly, the total amounts of non-contractile tissues, calculated as the sum of Triceps Surae IMAT and IMCT in both heads of the Gastrocnemius muscle, did not differ statistically in absolute terms (young: 56.96 cm^3^ ± 14.62 cm^3^ vs. older: 79.21 cm^3^ ± 26.08 cm^3^, p = 0.107, r = 0.54) but were significantly larger by 48.9% in the elderly cohort after normalization to Triceps Surae muscle volumes (young: 10.5% ± 2.1% vs. older: 17.3% ± 2.9%, p = 0.003, r = 0.83).

The measurements of tendon force demonstrated a 38.6% difference between young and older participants. Specific force, calculated as the ratio of tendon force and PCSA, was lower by 42.1% in the older cohort. Differences in specific force decreased to 29.6% when forces were normalized to the PCSA as calculated after subtraction of IMAT and IMCT from total muscle volume (Table 1). Correlational analyses demonstrated that the force output by the plantarflexor muscles was strongly correlated to specific force (r = 0.96, p < 0.001). However, the correlation between specific force and the cumulative relative amount of non-contractile materials within both heads of the Gastrocnemius muscle failed to reach statistical significance (r = -0.50, p = 0.142).

## Discussion

The present study aimed to establish reliable methods to quantitate the amount of intramuscular adipose and connective tissues, to test the sensitivity of these methods in detecting age-associated changes in the composition of the plantarflexor muscles and to determine their influence on the force generation capacity of this muscle group. Our findings demonstrate significant differences in both the amount of IMAT and IMCT, with the total content of Triceps Surae IMAT and the IMCT within the GM and GL muscles being greater in the elderly cohort by ~55% and ~49%, respectively. While in our study, the IMCT content within the SOL muscle could not be assessed due to time constraints in the data acquisition (overall duration of the scanning protocol ~90 min), these data reflect a ~50% increase in the overall amount of non-contractile tissues in the Triceps Surae complex.

Imaging and quantifying the IMAT content in the entire TS volume as opposed to a single cross-sectional image, as has been routinely reported earlier [16,17], as well as the focus on the 80+ years age group introduced several challenges. Previous studies have used fat- and water-suppressed images together to extract fat fractions [32]. While using image volumes with two such contrasts does result in greater classification accuracy and less sensitivity to intensity bias shading, it also increases scan time. Further, motion between scans can introduce image mismatch, which can also contribute to errors unless image alignment is used. In this study, the water saturated images were selected for IMAT segmentation based on the advantages that: (i) Muscle and connective tissue are both suppressed in this image while in a fat saturated images both fat and connective tissue present with very low/no intensity. Thus, the former enabled the isolation of fat voxels without confounding connective tissue. (ii) Contrast between fat and non-fat tissue was maximized, and (iii) Shading artifacts did not affect the fat voxel intensity since most of the fat voxels are distributed in the center of the image FOV. Care was taken to shim the entire imaging FOV to ensure that water intensity was uniformly suppressed across the imaging volume.

Several segmentation algorithms have been proposed for extracting fat from fat- and water-suppressed images [32,33]. Recently, Zhou and colleagues [33] proposed a Fuzzy C-means algorithm to segment water saturated or non-saturated images to quantify visceral abdominal fat from 6 axial slices. The authors established that the percentage of fat extracted from the water-saturated images had less variability than the non-saturated images. In the current paper, 3D bias correction was performed to reduce intensity shading artifacts. However, even with the bias correction, residual shading remained. Hence, the clustering was performed in 2D to enable a separate threshold for each image. The choice of the three clusters was based on the fact that due to the complex network of the fat voxels, a large number of voxels with overlapping fat and muscle exist (denoted as partial volume voxels). Using Fuzzy C-means clustering with two clusters tends to bias the cluster centroids, so that full voxel fat and muscle can be misclassified. Introducing a third cluster allows for the full fat and muscle voxels to be determined without bias introduced by the fairly large number of partial volume voxels. To validate the proposed approach for fat segmentation, fat fractions were additionally determined by IDEAL measurements in four subjects. Studies performed in rodents to validate the IDEAL technique have found excellent agreement of IDEAL results with those obtained through lipid extraction and qualitative and quantitative histologic analyses [34] and, therefore, represents the gold standard for fat/water quantitation. Since, in the first subjects examined in this study, the IDEAL sequence was not available on the scanner used in the present study, we based the quantification of fat on the segmentation of water-saturated FGRE images, and validated our approach against IDEAL measurements after this sequence had been installed on our scanner. The correlation of IMAT contents as determined from water-saturated FGRE images to those from IDEAL was high (r^2^ = 0.88), confirming that the proposed technique (image acquisition and segmentation algorithm) provides accurate estimates of the fat fraction. It should be noted that IDEAL is a specialized sequence and the current validation of the fat quantification from the more readily available water-saturated sequence will allow widespread application of MR based fat quantification.

The IMAT contents observed in our study showed moderate to good agreement with previously published data. In elderly subjects, Karampinos and colleagues [19] reported the IMAT content within the GM and SOL muscle to be ~6% and ~10%, respectively, as compared to the ~7% and ~9% found in our older cohort. The GL fat fraction, however, was considerably larger in our study (~7% vs. ~2%). As compared to data reported by Schwenzer et al. [35], the IMAT content of the SOL muscle agreed well in young subjects (~3% and ~4%, respectively) but was somewhat larger in our elderly subjects (6% vs. ~9%, respectively). In part, these differences may be attributable to the significantly older age of our older cohort (present study: ~83 yrs, Karampinos et al. [15]: ~62 yrs, Schwenzer et al. [35]: ~66 yrs), an explanation that supports our hypothesis, and to differences in the MRI procedures applied. Schwenzer et al. [35] applied a special multi echo sequence using a composite pulse (6 pulses) spectral-spatial excitation of lipids for muscular fat imaging, which was validated against reference spectroscopic techniques. The IDEAL sequence used by Karampinos et al. [19] computes fat fractions on a voxel-by-voxel basis, whereas our approach yields a binary image where voxels are considered to consist either entirely of fat or water. However, correlational analyses demonstrated a very good agreement of the results obtained by the two different approaches (r = 0.94) suggesting that Fuzzy C-Means clustering of water-saturated FGRE images may pose a good alternative for measurements of IMAT where IDEAL sequences are not available.

The dramatic age-associated increase of IMAT content observed in our study may be of fundamental clinical importance, as fatty infiltrations into the muscle are believed to be causally related to further muscle atrophy. While the MRI data obtained in the present study do not allow for conclusions about changes at the molecular level to be drawn, adipocytes and infiltrating macrophages have been shown to create an inflammatory environment through the secretion of adipokines and pro-inflammatory cytokines, such as TNF-α, IL-1 or IL-6 [36], which are likely to have multiple catabolic effects on skeletal muscles, e.g. by inhibiting the anabolic effects of IGF-1 or inducing a loss of myonuclei and satellite cells [37]. Adipose infiltrates in skeletal muscles are also believed to be responsible for impaired insulin sensitivity and excessive insulin secretion, which may hinder further cellular pathways associated with protein synthesis [38]. The resulting losses of contractile proteins are likely to trigger further accumulation of IMAT [39], thus perpetuating this process. In this context, we should point out that our subjects were not obese. The BMIs calculated for our young and elderly women were 20.0 ± 1.9 kg∙m^-2^ and 24.3 ± 1.7 kg∙m^-2^, respectively, which lies in the normal range reported for Asian cohorts [40]. Although longitudinal data have shown that IMAT inevitably increases during aging, irrespective of changes in body mass [4], *myosteatosis* and losses of intrinsic strength may be strongly exacerbated in sarcopenic obesity [41].

The intramuscular spatial distribution of adipose tissues may be of particular clinical relevance. Karampinos et al. [19] showed that fat compartmentalization is significantly different between post-menopausal women with and without Type 2 diabetes. They showed that, in diabetic patients, IMAT is preferentially accumulated in the center of muscle bellies rather than near the outer fascicle planes or in intermuscular regions. However, in boys with Duchenne muscular dystrophy, Marden et al. [42] showed that the fat infiltration is found along fascial planes even when there is minimal intramuscular fatty infiltration, suggesting that the former change may be an early indicator of disease. While the fat distribution within the muscle compartments was not extracted here, the current study shows that a significantly larger percentage of fat voxels are close to connective tissue in older subjects than in the younger age group. While any inferences are premature, this finding suggests that the intramuscular distribution of adipose tissues seen in sarcopenic muscles may resemble the one observed in Duchenne muscular dystrophy.

To the best of our knowledge, our study is the first to report, based on a novel MR imaging technique, quantitative data of the *in vivo* distribution of IMCT in skeletal muscles. The UTE sequence used for this purpose has been primarily applied to the study of cartilage compositional changes with osteoarthritis [43]. UTE-*T*_2_* imaging has been shown to correlate with the extent of cartilage matrix degeneration as measured by polarized light microscopy and composition analyses [44]. These UTE based imaging studies on the cartilage confirm that collagen characterization (organization and extent) is feasible with this method. The most direct validation of UTE imaging for collagen deposition comes from a study of myocardial fibrosis performed in rodents [45], which demonstrated that the areas of fibrosis as identified via UTE imaging corresponded with collagen-rich areas observed in histology. The current study is the first to use UTE- *T*_2_* to map connective tissue, which is a collagen rich tissue. It is noteworthy that the UTE sequence used in the current paper is very similar to that used in the myocardial fibrosis study. In contrast to the myocardial fibrosis study, however, we employed calculated *T*_2_* maps (instead of subtracted images) which is a quantitative technique and reduces the effect of shading artifacts and improves the segmentation of connective tissue.

The analyses of the UTE images demonstrate that the relative amount of collagenous tissues within both the GM and GL was considerably larger in our older cohort (~50% difference in total IMCT content), with the between-group differences being somewhat larger in the GL than in the GM. Collagenous tissues play an important role in maintaining the structural integrity of skeletal muscles and their supporting tissues [46], and in the transmission of contractile forces onto the skeleton [47]. Quantification of these connective tissue within the muscle has not been possible till now with imaging methods because, as mentioned before, these are semi-solid structures and manifest as signal void in routine MRI unless the relatively sophisticated technique of UTE MR imaging is utilized. In this technique, the MR signal has to be collected a thousand times faster (μs compared to ms) than in routine MRI, thus rendering it significantly more hardware- and software-intensive. Cartilage, cortical bone, plaques and other solid and semi-solid structures (including the IMCT) within the human body are then registered as hyperintense structures. Most measurements of collagen content by other groups on the other hand, have been performed on biopsies, typically in animal models for longitudinal studies, with microscopic assessment of stained muscle cross-sections. Early animal experiments performed in rodents have demonstrated [48] that the aging process may bring about significant increases in IMCT content, with the endomysium being particularly affected. Recent research suggests that the increasing accumulation of IMCT may be related to a decline of function of satellite cells, which tend to convert from a myogenic to a fibrogenic lineage at older age [49,50]. These findings reflect the impaired regenerative capacity of elderly muscles, in which damaged contractile proteins may be displaced by collagenous infiltrations. While the functional consequences of increasing muscular fibrosis are not yet clearly understood, it has been suggested that excessive IMCT accumulation accompanied by age-associated collagen modifications might increase the stiffness of the extracellular matrix formed by connective tissues [51], which might further impair muscle function [52]. In the future, modeling-based studies from this group will help test this hypothesis and further elucidate functional implications of age-associated muscle fibrosis as well as fatty infiltration.

We further aimed to estimate the effects of age-associated increases in IMAT and IMCT content on the intrinsic force generation capacity of the plantarflexor muscles. Due to the relatively large variance of force recordings and the small sample investigated, the differences in our measures of muscle volumes, plantarflexor force and specific force failed to reach statistical significance. On average, however, we found the Triceps Surae volume to be smaller by ~20% and plantarflexor force to be lower by ~40% in the older cohort. These results agree well with previous findings [5,6] and testify to the fact that age-associated losses in muscle volume are accompanied by disproportionate losses in muscle strength. In our pilot study, no statistical correlations between specific force and the total relative amount of non-contractile materials within the Triceps Surae complex could be established. While this lack of significance may partly be due to our limited sample size, several factors known to strongly affect muscle quality, such as muscle architecture, fiber type distribution and single-fiber specific tension, have not been accounted for in the present survey. Most importantly, differences in the neural drive to muscles [53-56] may have influenced our measurements of specific force. Of particular relevance to the present study, research suggests that aging may be associated with impaired intermuscular coordination, as characterized by excessive antagonist co-activation [57]. Also, methodological challenges in determining the plantarflexor strength, such as the inevitable misalignment of the ankle’s center of rotation and the dynamometer’s axis of rotation during MVC [58,59], may have introduced bias. Subtracting the amount of non-contractile tissues from the total volume of the Triceps Surae complex, however, reduced the age-associated differences in specific force from ~42% (when forces were normalized to the entire Triceps Surae volume) to ~30%, which supports the hypothesis that accumulation of intramuscular adipose and connective tissues represents an important determinant of loss of specific force at older age.

## Conclusions

In conclusion, using novel MR imaging and analyses techniques, we were able to determine the intramuscular amounts of both adipose and connective tissues in the plantarflexor muscles of young and elderly women. Our pilot data showed significant age-associated differences in both IMAT and IMCT content but no statistical correlation between these non-contractile materials and specific force, representing the plantarflexor muscle quality. In the future, within our group, data on the amount and spatial distribution of both contractile and non-contractile tissues (including IMAT and IMCT) will be integrated into subject-specific, multi-scale computational models of skeletal muscles, to help elucidate the functional roles of the material properties of these different tissues and how they affect strength in sarcopenia and other pathologies [60]. Combined with future, more complete studies carried out in larger samples and assessing further factors known to affect muscle quality, such as neural drive, a more robust relationship is likely to be established between muscle composition and intrinsic strength and potentially allow subject-specific tailor-made exercise paradigms to be devised to alleviate the debilitating factors of aging.

## Abbreviations

BW: Bandwith; CT: Computed tomography; DXA: Dual-energy X-ray absorptiometry; FA: Flip angle; FGRE: Fast gradient recalled echo MRI sequence; IDEAL: Iterative decomposition of water and fat with echo asymmetry and least-squares estimation; IMAT: Intramuscular adipose tissue; IMCT: Intramuscular connective tissue; MA: (Achilles tendon) moment arm; MRI: Magnetic resonance imaging; MVC: Maximum voluntary contraction; NEX: Number of excitations; PCSA: Physiological cross-sectional area; SNR: Signal-to-noise ratio; TE: Echo time; TR: Repetition time; UTE: Ultra-short echo time MRI sequence.

## Competing interests

There are no competing interests.

## Authors’ contributions

RC: Main investigator, drafting and revision of the manuscript. VM: Data acquisition and analysis. US: Data analysis and interpretation, revision of the manuscript. JD: Data acquisition. SS: Project supervisor, study design, data acquisition, revision of the manuscript. All authors read and approved the final manuscript.

## Pre-publication history

The pre-publication history for this paper can be accessed here:

http://www.biomedcentral.com/1471-2474/15/209/prepub
